# Engineering broad-spectrum phage-resistant *Escherichia coli* via adaptive and programmable defense strategies

**DOI:** 10.1128/aem.01596-25

**Published:** 2025-10-22

**Authors:** Zhenwen Xu, Yunfei Huang, Yuqi Dong, Xiaoping An, Yigang Tong, Mengzhe Li

**Affiliations:** 1 BAICSM, State Key Laboratory of Green Biomanufacturing, College of Life Science and Technology, Beijing University of Chemical Technology 627792, Beijing, China; Universidad de los Andes, Bogotá, Colombia

**Keywords:** fermentation, phage contamination, phage-resistant strain, spontaneous mutation, CRISPR/Cas9

## Abstract

Phage contamination, which impacts product quality and production efficiency, remains a major challenge in industrial fermentation. Although bacteria have evolved various defense systems to combat phage infection, these systems often suffer from narrow host specificity and limited efficacy. In this study, we isolated and characterized a novel lytic *Escherichia coli* phage, TR2, from a contaminated fermentation substrate. Its strong environmental stability, short latency period, and high lytic activity render it a significant threat to fermentation processes. Genomic sequencing revealed that phage TR2 has a linear, double-stranded DNA genome of 45,171 bp with a G+C content of 44% and 74 coding sequences. On the basis of the physiological characteristics and genomic features of this phage, we developed two strategies to generate phage-resistant *E. coli* strains: (i) selection of spontaneous mutations in bacterial surface receptors to prevent phage adsorption and infection and (ii) integration of an exogenous CRISPR/Cas9 system to confer sequence-specific immunity. Spontaneous mutation provides broad-spectrum resistance but at the cost of fitness and evolutionary stability, whereas CRISPR/Cas9 ensures long-term, programmable immunity with minimal growth defects. Importantly, both strategies successfully protected bacterial cultures from phage infection without compromising recombinant protein production, highlighting their potential for industrial application. Our findings provide a practical approach for mitigating phage contamination in industrial fermentation processes. This study also highlights the advantages and limitations of spontaneous mutations and natural phage defense systems, offering valuable insights for the design of more effective phage-resistant microbial platforms.

**IMPORTANCE:**

Phage contamination is a significant challenge in industrial fermentation and severely impacts product quality and production efficiency. We systematically compared spontaneous mutation and CRISPR/Cas9-mediated immunity as two strategies for engineering phage-resistant *E. coli* strains. Both approaches effectively protected bacterial cultures from phage infection without compromising recombinant protein production, underscoring their potential for industrial applications. Notably, spontaneous mutation conferred broad-spectrum resistance but was associated with fitness costs and limited evolutionary stability. In contrast, CRISPR/Cas9-based immunity offered long-term, programmable protection with minimal growth impairment. By delineating the trade-offs between these two strategies, our work provides a framework for selecting tailored phage resistance solutions suited to diverse biomanufacturing scenarios.

## INTRODUCTION

Phage contamination in biotechnology, particularly in fermentation processes, presents a significant challenge to the industry. Phages, viruses that specifically infect bacteria, can severely disrupt industrial-scale fermentation by reducing production yields and contaminating products (1). This not only compromises the quality of the final product but also leads to substantial economic losses (2). Current strategies to address phage contamination in industrial fermentation largely rely on the use of chemical treatments or physical methods to remove or inactivate phages (3). However, these approaches are often inefficient, costly, or disruptive to the overall fermentation process. Therefore, it is imperative to develop more efficient and sustainable strategies to prevent and control phage outbreaks within biotechnological applications.

To combat phage invasion, bacteria have developed a wide range of defense systems, many of which have been harnessed for biotechnological purposes. These systems include the restriction modification system (4), CRISPR/Cas system (5), abortive infection system (6), phage exclusion system (1), and toxin and antitoxin system (7). The restriction-modification system acts as a bacterial immune system, identifying and cleaving foreign DNA from invading phages (4). The abortive infection system, in turn, sacrifices the host bacteria to prevent phage replication (6). The phage exclusion system works by blocking phage attachment (1), whereas toxin‒antitoxin systems regulate bacterial stress responses, ensuring cellular survival under phage attack (7).

Among these defense mechanisms, the CRISPR/Cas system stands out as a particularly versatile tool. The CRISPR/Cas9 system, a representative type II-A CRISPR/Cas system (8), has been widely used in genetic engineering for precise genome editing. This system allows bacteria to integrate DNA fragments from previous phage infections into their genome, providing adaptive immunity (9). Upon reinfection by the same phage, bacteria produce crRNA that guides the Cas9 protein to the viral DNA, leading to its degradation (10, 11). The precision and adaptability of the CRISPR/Cas9 system make it a valuable tool for both basic research and industrial applications, enabling targeted resistance to specific phages.

Other effective defense strategies involve the alteration or masking of bacterial surface receptors and the use of decoy receptors to prevent phage attachment (12). This approach is simple, straightforward, and effective in allowing bacteria to evade phage infection without necessitating complex genetic alterations (13). Bacteria that modify their surface structures can rapidly adapt to phage attacks, making this an advantageous strategy for short-term resistance in fluctuating environments (14).

In this study, we isolated a novel lytic *Escherichia coli* phage, designated TR2, from contaminated fermentation samples. This phage efficiently infected the industrially relevant strain *E. coli* BL21 (DE3), severely disrupting the fermentation process. Through comprehensive characterization and genomic analysis of TR2, we constructed two types of engineered phage-resistant *E. coli* strains: one utilizing spontaneous mutations in phage receptor genes to block adsorption and the other employing a CRISPR/Cas9-based targeted immune system. We systematically compared their phage inhibition efficiency, broad-spectrum resistance, and genetic stability. This work provides theoretical support and practical guidance for the development of efficient and stable phage-resistant strains, offering a feasible solution for controlling phage contamination in industrial fermentation systems.

## RESULTS

### Morphology of *E. coli* phage TR2

**Fig 1:**
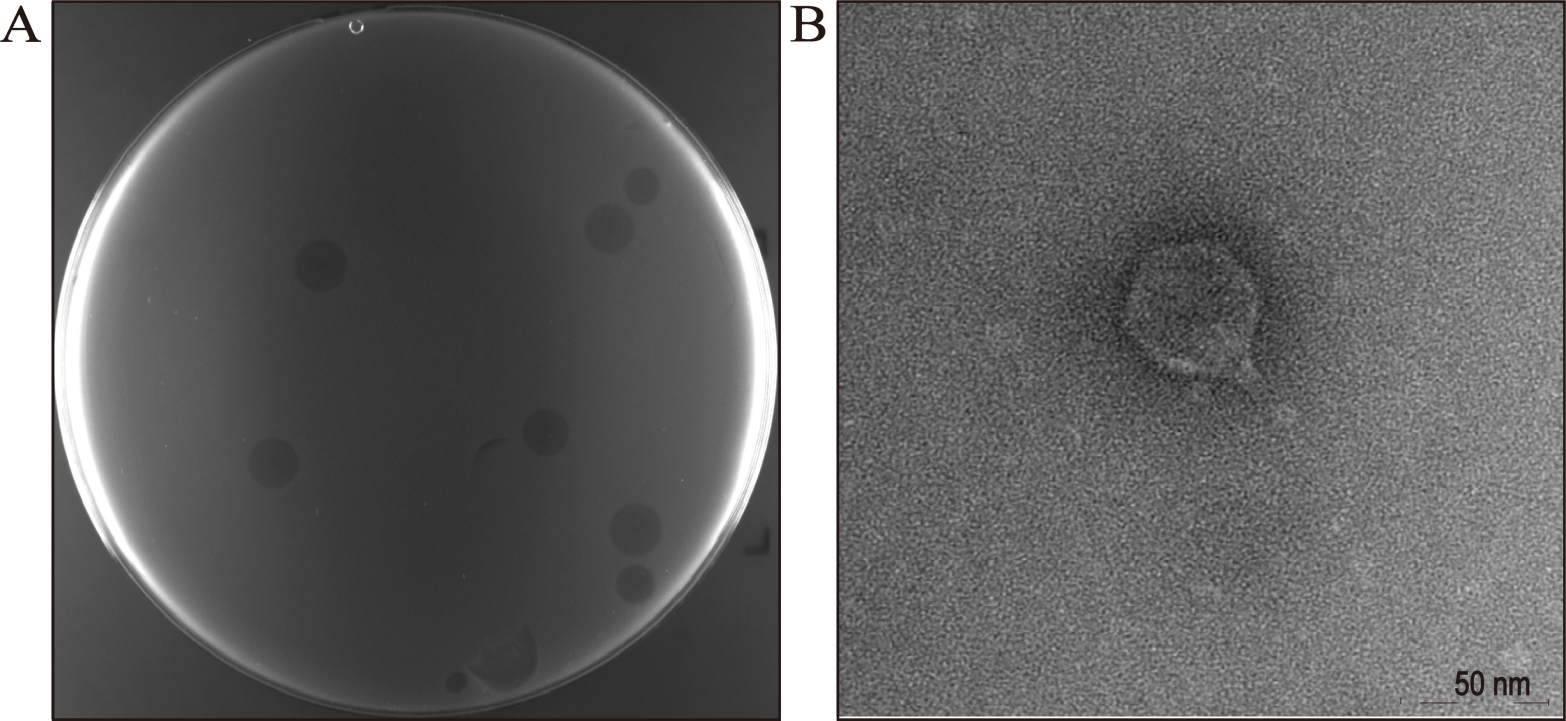
Plaques and microscopic morphology of phage TR2. (**A**) Plaques formed by phage TR2 on the surface of the lawn of wild-type BL21(DE3). (**B**) Transmission electron micrograph of phage TR2.

BL21(DE3), a widely used model microorganism in industrial fermentation, served as the host for phage isolation. A lytic phage, designated TR2, was successfully isolated from the fermentation substrate. It formed circular, transparent plaques with an average diameter of 6.42 ± 1.32 mm on the wild-type BL21(DE3) bacterial lawn (Fig. 1A). Transmission electron microscopy (TEM) analysis revealed that TR2 possessed a capsid with a diameter of 50 nm and a short tail that was 10 nm in length, resulting in a head-to-tail ratio of 500% (Fig. 1B). The TEM image of phage TR2 revealed a typical podovirus morphology.

### Biological characterization of phage TR2

The host range of phage TR2 was evaluated against 27 different bacterial strains using the efficiency of plating (EOP) method. The results demonstrated that phage TR2 exhibited lytic activities exclusively against *E. coli* strains. In contrast, it failed to lyse *Pseudomonas aeruginosa*, *Klebsiella pneumoniae*, *Proteus mirabilis*, *Acinetobacter baumannii*, *Enterobacter cloacae*, and *Staphylococcus aureus*. Notably, among the 12 *E. coli* strains, TR2 displayed strong specificity, targeting only BL21(DE3) and DH5α while being ineffective against the other tested *E. coli* strains (Table S1).

**Fig 2:**
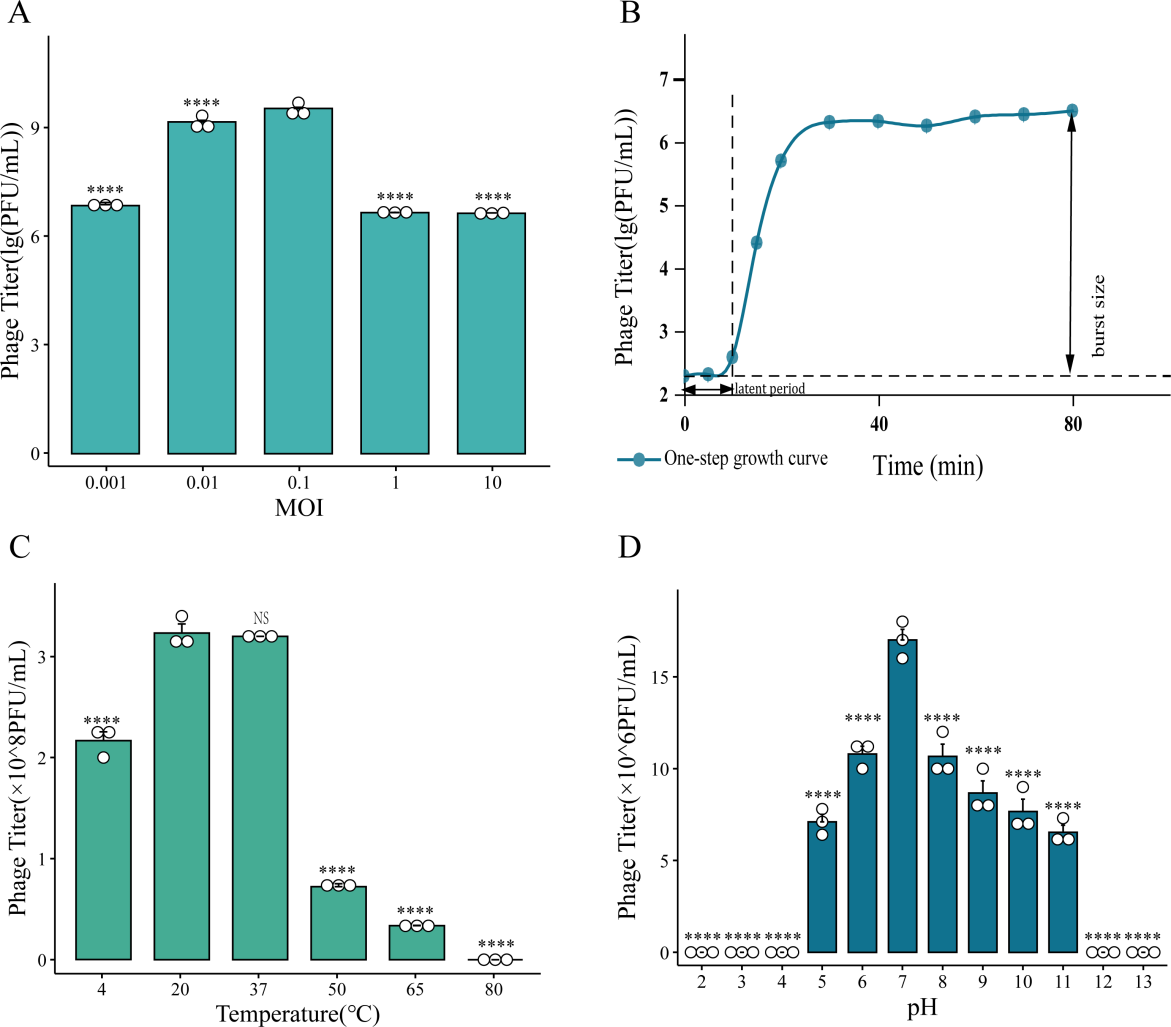
Biological characteristics of phage TR2. (**A**) Optimal multiplicity of infection for phage TR2. (**B**) One-step growth curve of phage TR2 under the optimal multiplicity of infection. (**C**) Thermal stability of phage TR2. (**D**) pH stability of phage TR2.

To determine the optimal multiplicity of infection (MOI) for phage TR2, BL21(DE3) cells were infected at MOIs of 10, 1, 0.1, 0.01, and 0.001. After 6 h of infection, the highest phage titer of 3.5 × 10^9^ PFU/mL was observed at an MOI of 0.1, indicating that 0.1 was the optimal MOI (Fig. 2A). We subsequently constructed a one-step growth curve of phage TR2 at the optimal MOI (Fig. S2), and this analysis revealed a short latency period of 10 min and an average burst size of 77 PFU/cell (Fig. 2B), highlighting the rapid replication and strong lytic ability of this phage.

The environmental stability of phage TR2 was investigated under different pH and temperature conditions. TR2 exhibited substantial tolerance to acidic and alkaline environments, maintaining over 50% of its activity after 1 h of exposure to pH 5.0–11.0 (Fig. 2C and D; Fig. S1). However, the phage was nearly completely inactivated at pH ≤4 or pH ≥12 (Fig. 2C and D). Thermal stability assessment indicated that the phage titer did not significantly change under exposure to temperatures ranging from 20 to 37°C for 30 min. At temperatures above 50°C, phage activity significantly decreased in a temperature-dependent manner.

### Genomic features and phylogenetic analysis of TR2

**Fig 3:**
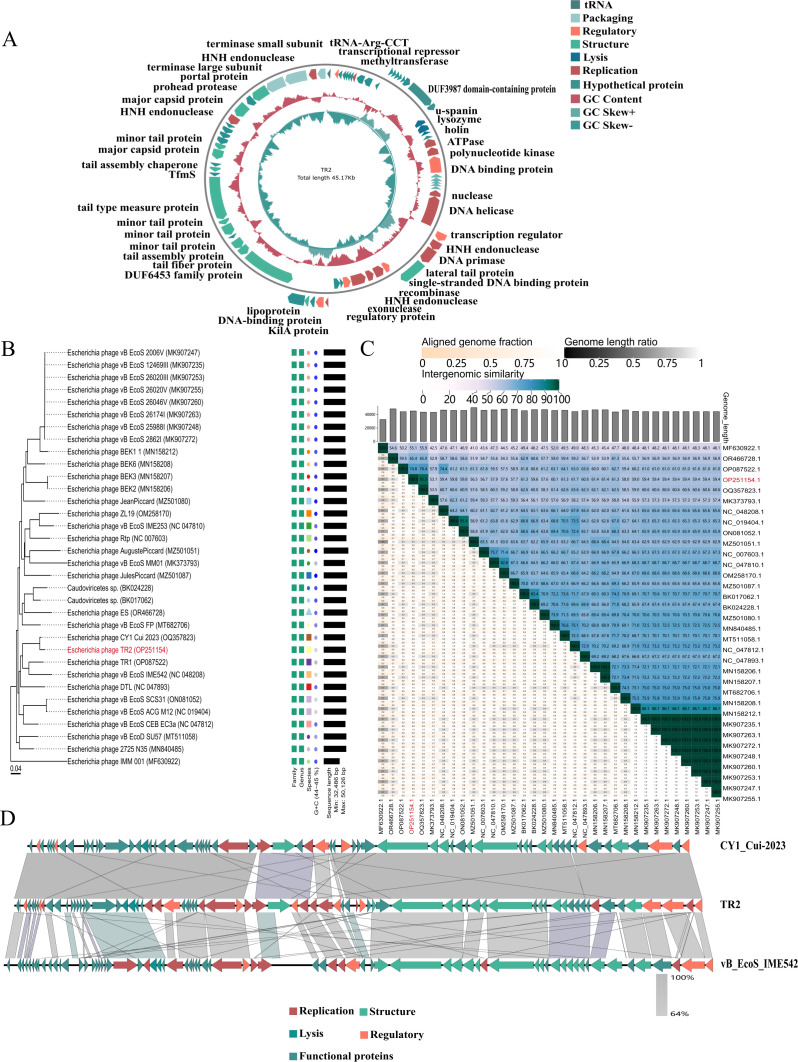
Characterization and comparative analysis of the phage TR2 genome. (**A**) The circular genetic map of phage TR2, including the GC content and GC skew, is illustrated in the inner circle. A total of 74 open reading frames (ORFs) of phage TR2 are displayed in the outermost circle and are categorized using different colors. (**B**) A phylogenetic tree was constructed on the basis of the complete genome sequence of the phage to demonstrate the evolutionary relationship of phage TR2 with 36 other *E. coli* phages. (**C**) Average nucleotide identity (ANI) among phages was calculated using the VIDIRIC online tool. The results are presented using a heatmap, with phage TR2 highlighted. (**D**) Comparative genomic analysis of phage TR2: arrows are utilized to visualize transcriptional directionality, and different functional ORFs are distinguished by various colors.

The TR2 genome is a linear double-stranded DNA with a total length of 45,171 bp and a G+C content of 44% (Fig. 3A). The complete genome of phage TR2 contains 74 predicted open reading frames (ORFs), 43 of which have defined or putative functional annotations, whereas 31 are classified as hypothetical proteins. Genomic analysis confirmed the absence of antibiotic resistance genes, virulence factors, and lysogenic elements. On the basis of functional annotations, these ORFs can be grouped into seven major functional modules, including structural components, DNA packaging, replication, lysis, regulation, tRNA-related functions, and hypothetical proteins. Representative genes within each module, such as the large and small terminase subunits (ORF72/ORF74), major capsid proteins (ORF61/ORF68), holin (ORF23), lysozyme (ORF22), and spanin (ORF21), were predicted on the basis of sequence homology. The complete functional annotations of these genes are provided in Table S2.

Comparative analysis using the online tool BLASTN revealed that phage TR2 presented the highest homology with the *Escherichia* phage CY1_Cui-2023 (GenBank accession number: OQ357823.1), exhibiting 92% genome coverage and 99.69% sequence identity. The phylogenetic tree was constructed on the basis of the complete genome sequences of 37 phages that shared high homology with phage TR2 (Fig. 3B). Phage TR2 was clustered in the same branch as the *Escherichia* phage CY1_Cui-2023. According to the VICTOR classification, all phages in this branch belonged to the same family, *Drexlerviridae*, and subfamily, *Braunvirinae*.

The average nucleotide identity (ANI) among the 37 phage genomes was calculated and visualized using a heatmap to illustrate the genomic similarity (Fig. 3C). Compared with phage TR2, *Escherichia* phage CY1_Cui-2023 presented the highest ANI value of 95.5%. *Escherichia* phages CY1_Cui-2023 and vB_EcoS_IME542 (GenBank accession number NC_048208.1) were employed for comparative genomic analysis because of their high sequence similarity with phage TR2 (Fig. 3D). The three phages shared highly similar conserved frameworks in their structural proteins. The genome of phage TR2 was found to lack antibiotic resistance genes, virulence factors, and lysogenic elements, indicating favorable biosafety characteristics. Additionally, analysis using the PhageTerm tool revealed that TR2 adopts a canonical Pac-type DNA packaging mechanism (headful mechanism), with the packaging initiation site located at nucleotide position 18,637 on the plus strand.

### Mutation analysis of spontaneously mutated phage-resistant strains

**Fig 4:**
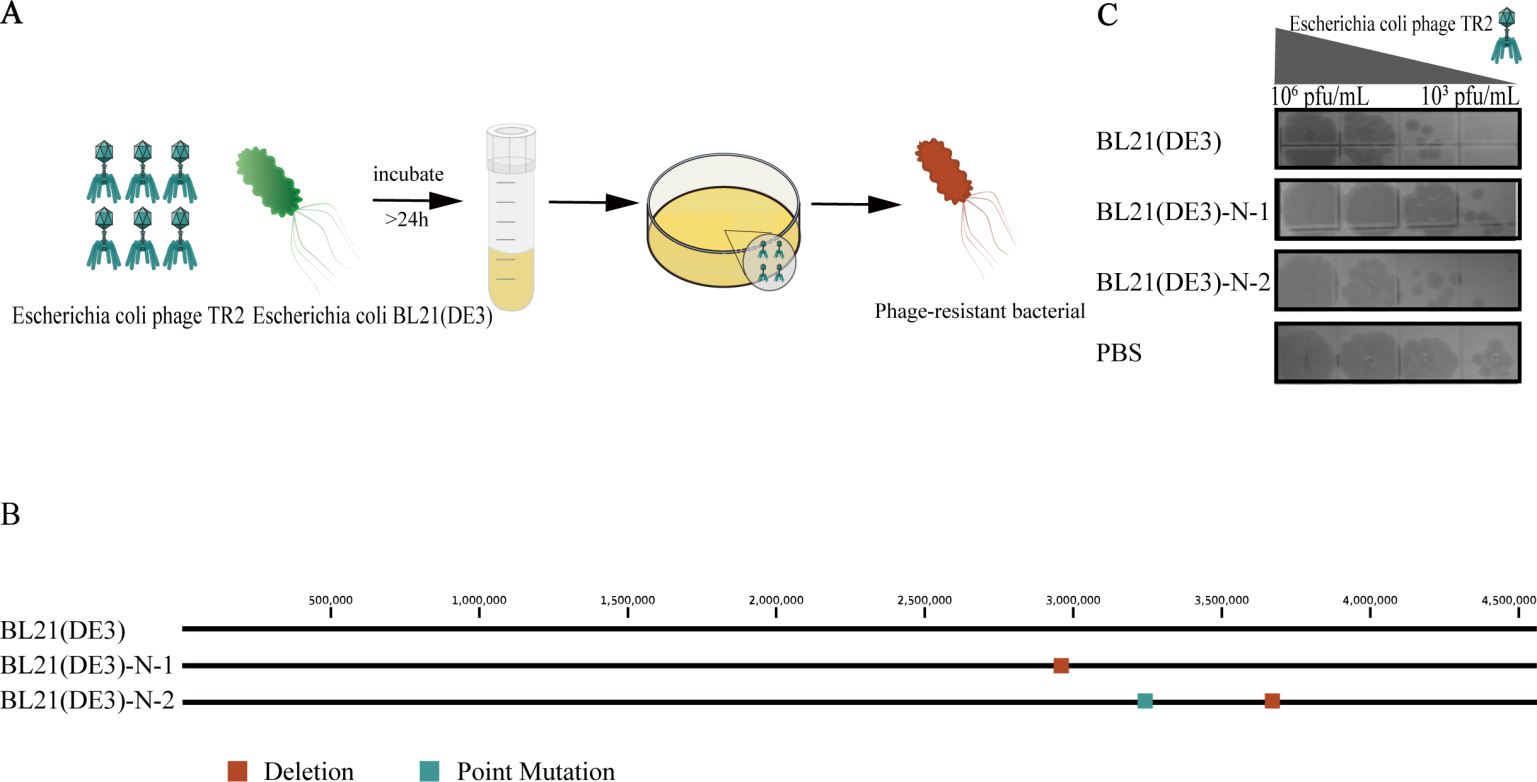
Preparation and identification of spontaneously mutated phage-resistant strains. (**A**) Schematic illustration of the experimental design for preparing spontaneously mutated phage-resistant strains, with mutant and wild-type BL21(DE3) strains differentiated by distinct colors. (**B**) Identification of mutation sites and mutation types in the engineered resistant strains. (**C**) Adsorption capabilities of different phages on wild-type BL21(DE3) and its mutants.

To investigate the genetic basis of resistance to phage TR2, whole-genome sequencing was performed on two spontaneously mutated phage-resistant strains: BL21(DE3)-N-1 and BL21(DE3)-N-2. Mutation analysis revealed distinct genetic alterations in these strains (Fig. 4B). In BL21(DE3)-N-1, a 777 bp deletion disrupted an ISIA family transposase and the 2-C-methyl-D-erythritol 4-phosphate cytidylyltransferase (ISPD) gene (15). In BL21(DE3)-N-2, two mutations were identified: a point mutation (A→G) in the gene encoding the LPS transporter protein LPTA (16) and a base deletion in the gene encoding LPS 1,2-glucosyltransferase, a core enzyme in LPS synthesis (17). Genomic analysis indicated that the phage resistance of BL21(DE3)-N-2 may result from mutations in the LPTA gene and the gene encoding LPS 1,2-glucosyltransferase, an enzyme involved in lipopolysaccharide (LPS) biosynthesis. These alterations may have modified the structure or distribution of surface receptors, thereby impairing phage recognition and adsorption. In contrast, although mutations in the ISIA transposase and  ISPD genes were identified in BL21(DE3)-N-1, there is currently no direct evidence linking these mutations to changes in receptor structure. Furthermore, adsorption assays confirmed significantly reduced or abolished phage TR2 adsorption in both mutants compared with that in wild-type BL21(DE3) (Fig. 4C). These results suggest that spontaneous mutations in key receptor-associated genes can impair phage adsorption, allowing bacteria to evade phage infection through receptor site modification or loss.

### Antiphage activities in phage-resistant strains

**Fig 5:**
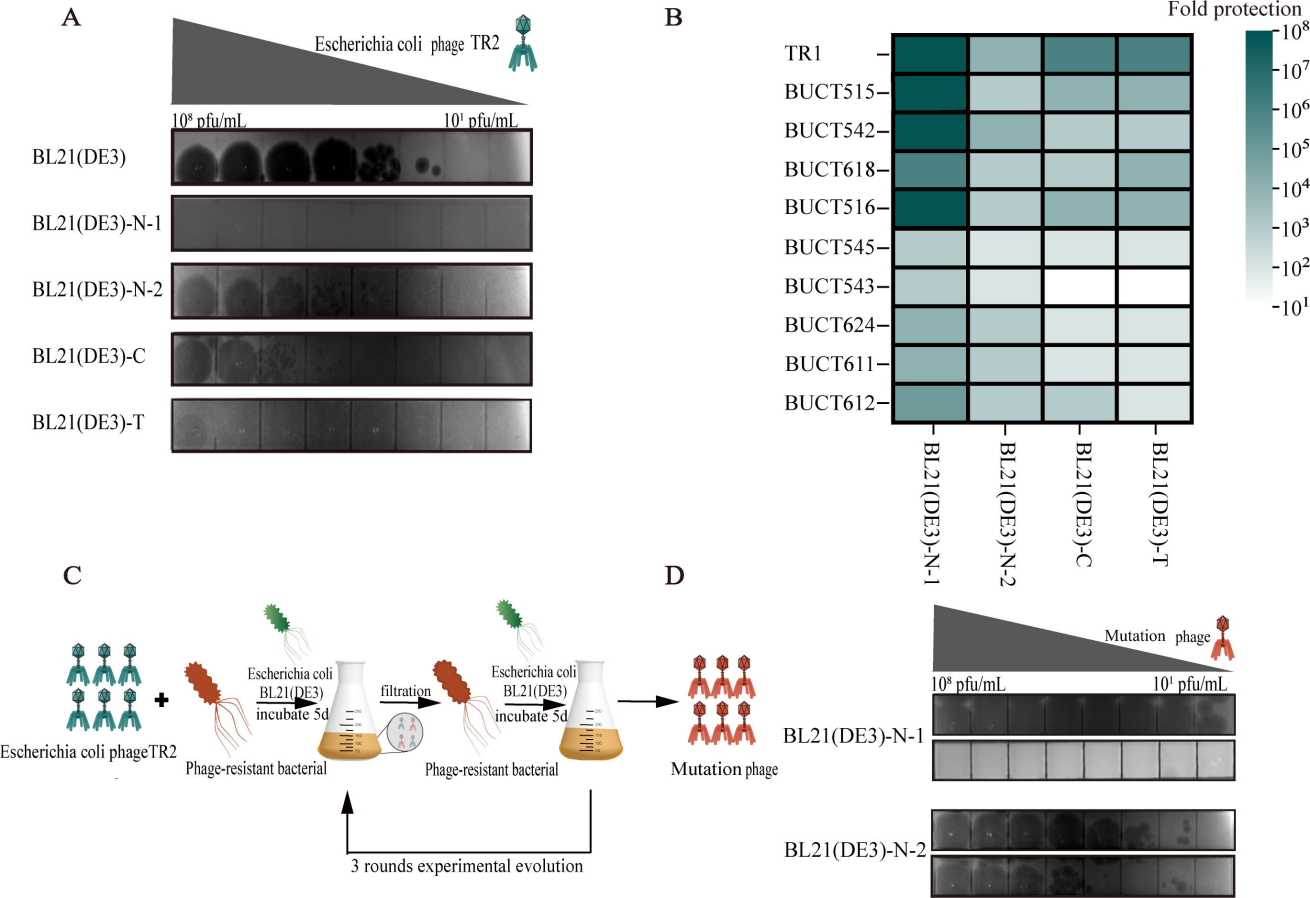
Phage resistance activities of spontaneously mutated phage-resistant strains and engineered CRISPR/Cas9 recombinant strains. (**A**) EOP assay to confirm the TR2 phage resistance activity of spontaneously mutated phage-resistant strains and the engineered CRISPR/Cas9 recombinant strain. (**B**) Evaluation of the broad-spectrum antiphage potential of spontaneously mutated phage-resistant strains and engineered CRISPR/Cas9 recombinant strains. (**C**) Schematic illustration of the experimental setup for stability evaluation of phage resistance, with mutant and wild-type TR2 differentiated by distinct colors. (**D**) Comparative analysis of plaque formation efficiency between wild-type and evolved phage strains on host bacterial lawns.

To assess phage resistance, we compared the antiphage activities of spontaneously mutated phage-resistant strains and CRISPR/Cas9-engineered recombinant strains. EOP assays demonstrated that both strategies conferred varying degrees of phage resistance to wild-type BL21(DE3) (Fig. 5A). When tested against a panel of ten phages, BL21(DE3)-N-1 demonstrated broad-spectrum resistance, reducing infection by more than four orders of magnitude against eight phage strains (Fig. 5B). In contrast, the CRISPR/Cas9 strains displayed strong but narrow protection, notably showing enhanced resistance to the phage TR1, which shares high sequence similarity with TR2. These results suggest that spontaneously mutated phage-resistant strains possess broader antagonistic spectra against diverse phage populations, whereas engineered CRISPR/Cas9 recombinant strains provide strong but phage-specific protection.

To evaluate the stability of phage resistance, we performed three successive coculture cycles. After three cycles of adaptive coevolution with the spontaneously mutated phage-resistant strains BL21(DE3)-N-1 and BL21(DE3)-N-2, the evolved phage variants TR2-E-1 and TR2-E-2 were isolated, respectively (Fig. 5D). TR2-E-1 and TR2-E-2 shared the same nonsynonymous mutation, G24084A, in ORF50 (tail tip protein). In contrast, no phage escape variants were observed after three cycles of coculture with engineered CRISPR/Cas9 recombinant strains, indicating that engineered CRISPR/Cas9 recombinant strains provide more stable and long-lasting defense against phage infections.

### Phage resistance, growth inhibition, and recombinant protein production

**Fig 6:**
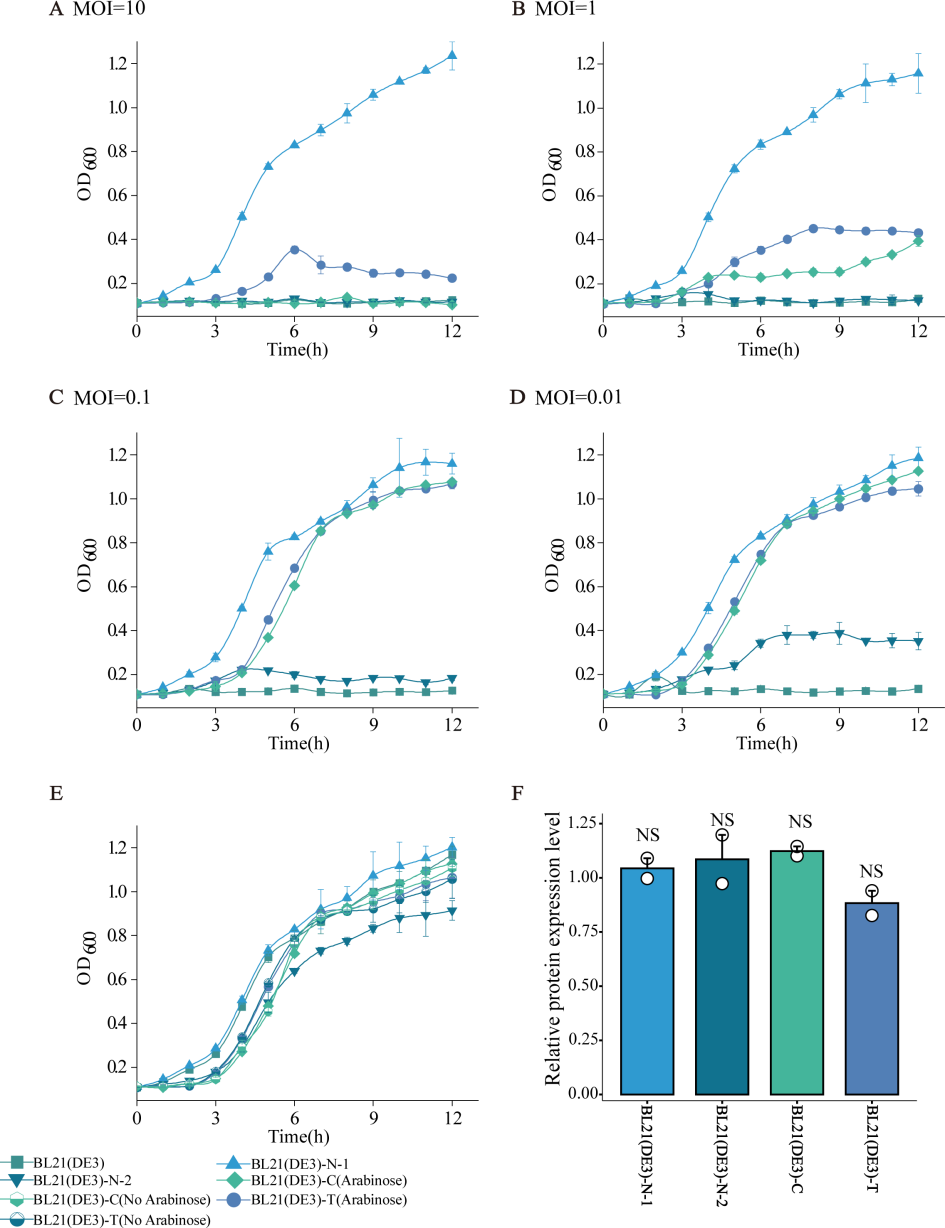
Growth curves of recombinant strains treated with phage TR2 at the indicated multiplicities of infection (MOIs). The recombinant strains not treated with phage or wild-type BL21(DE3) at the respective MOIs were used as control groups. (**A**) MOI = 10. (**B**) MOI = 1. (**C**) MOI = 0.1. (**D**) MOI = 0.01. (**E**) Growth curves of the recombinant strains not treated with phage and without induced expression of the CRISPR/Cas9 system. (**F**) Densitometric quantification of protein expression levels was performed. Band intensities were analyzed using ImageJ software and normalized to the wild-type BL21 (DE3) expression level, which was set to 1.0.

To evaluate resistance performance under phage pressure, we monitored the growth of spontaneously mutated phage-resistant strains and engineered CRISPR/Cas9 recombinant strains under phage TR2 challenge at various MOIs (Fig. 6A through D). Both engineered CRISPR/Cas9 recombinant strains exhibited significant resistance to phage TR2 at MOIs of 1, 0.1, and 0.01, although their growth was moderately inhibited in an MOI-dependent manner. Among the spontaneously mutated strains, BL21(DE3)-N-1 showed complete resistance across all MOIs tested, including an MOI of 10, whereas BL21(DE3)-N-2 exhibited pronounced growth suppression. These results confirm that both strategies confer phage resistance, with BL21(DE3)-N-1 offering the most robust and stable protection under high phage pressure. To assess the potential fitness costs associated with phage resistance, bacterial growth was evaluated under phage-free conditions (Fig. 6E). The growth of BL21(DE3)-N-1 was comparable to that of wild-type BL21(DE3), suggesting that there was no detectable fitness burden. However, BL21(DE3)-N-2 showed reduced growth, with its OD_600_ reaching only 78.25% of that of the control after 12 h of incubation. The growth of the CRISPR/Cas9 recombinant strain was also slightly compromised, with an OD_600_ approximately 90% of that of the control. In addition, induction of the CRISPR/Cas9 system had a minimal effect on cell growth, with a less than 2% difference between induced and uninduced cultures, indicating a low metabolic burden.

Finally, we examined the effects of these resistance strategies on recombinant protein production. Using a T7 expression system, we constructed the plasmid pET28a (+) −603helicase (GenBank: QVW29510.1) to express phage DNA helicase protein (approximately 49.02 kDa). SDS‒PAGE and western blot analysis revealed that recombinant protein production in the spontaneously mutated phage-resistant strains and engineered CRISPR/Cas9 recombinant strains was comparable to that in the wild-type BL21(DE3) strain (Fig. 6F; Fig. S3). These results indicated that neither resistance strategy adversely affects the recombinant protein production capacity. These findings underscore that both strategies successfully confer resistance to phage infection while maintaining bacterial growth and recombinant protein yield, demonstrating their viability for industrial applications.

## DISCUSSION

Phage contamination remains a critical challenge in industrial fermentation, compromising production quality and leading to significant economic losses (18). Therefore, developing phage-resistant bacterial strains is a promising alternative strategy. In the ongoing evolutionary “arms race” between bacteria and phages, bacteria have evolved multiple defense mechanisms (19). Among them, the CRISPR/Cas9 system has been repurposed as a genetic tool for precise genome editing while retaining its original function as an adaptive immune defense (20). Additionally, bacteria can develop phage resistance through spontaneous mutations that alter, mask, or eliminate phage receptors, rendering them unrecognizable to invading phages (21).

In this study, we first isolated and characterized phage TR2, a lytic *E. coli* phage with strong environmental stability, a short latency period, and high lytic activity, which render it a significant threat to fermentation processes (Fig. 2) (2). Genomic sequencing of TR2 yielded crucial insights into its genetic composition and established a foundation for the development of programmable phage resistance strategies. To counteract TR2 contamination, we successfully constructed phage-resistant BL21(DE3) strains via two distinct strategies: (i) spontaneous mutation, where bacteria acquire phage resistance through receptor modifications, and (ii) CRISPR/Cas9-mediated immunity, which provides targeted genetic defense.

Both spontaneously mutated phage-resistant strains and engineered CRISPR/Cas9 recombinant strains exhibited strong resistance to phage TR2 (Fig. 5A). The spontaneous mutant BL21(DE3)-N-1 displayed the highest phage resistance, as evidenced by the complete absence of plaques. However, the identified mutation sites (IS1A transposase and ISPD genes) have no clearly established connection to phage receptors, and the underlying mechanism requires further investigation. Moreover, this strategy is limited by its random nature, meaning that different mutations may confer varying levels of resistance (22). In contrast, the CRISPR/Cas9 system provides targeted phage resistance by precisely recognizing and degrading phage DNA (11). While the engineered CRISPR/Cas9 recombinant strains were also resistant to TR2, they exhibited lower efficiency in broad-spectrum phage defense, as their protection was sequence specific (Fig. 5B) (5). This highlights a trade-off: spontaneous mutations may offer broader protection but are less predictable, whereas CRISPR/Cas9 offers stable and programmable immunity; however, the targeted design of specific phages may be necessary.

Despite conferring resistance, both strategies had varying impacts on bacterial growth (Fig. 6E) (23, 24). BL21(DE3)-N-2 exhibited a significant growth delay, suggesting that receptor mutations may interfere with essential bacterial functions, such as nutrient uptake or membrane stability (25). The engineered CRISPR/Cas9 recombinant strains, while slightly compromised, maintained better growth fitness than spontaneous mutants did. Interestingly, the expression of the CRISPR/Cas9 system did not impose a significant metabolic burden, as there was no difference between the induced and uninduced strains (Fig. 6E). This finding contrasts with some previous reports in which maintaining CRISPR-mediated defense systems required high-energy expenditure (26). This discrepancy may be due to differences in plasmid expression levels or the metabolic adaptability of *E. coli* BL21(DE3). These results suggest that spontaneous mutations may compromise bacterial growth due to unintended disruptions and that CRISPR/Cas9-based strains retain greater growth efficiency, making them a more viable option for large-scale fermentation.

To assess the long-term effectiveness of both strategies, we conducted phage resistance stability assays over multiple culture cycles. After three rounds of coculture, phage TR2 regained the ability to infect the spontaneous mutant strains (Fig. 5D), indicating that the receptor-based resistance mechanism may drive the arms race between bacteria and phages. This aligns with previous findings showing that phages can circumvent receptor modifications in the host cell by undergoing mutations in the tail fiber, thereby maintaining their ability to infect the host (27). In contrast, no phage escape mutants were observed in the engineered CRISPR/Cas9 recombinant strains, suggesting that sequence-based immunity is more robust and stable over time. This finding is significant, as it demonstrates that engineered CRISPR/Cas9 recombinant strains are less susceptible to phage adaptation, making it a more reliable strategy for long-term industrial applications.

A key requirement for industrial *E. coli* strains is their ability to efficiently express recombinant proteins while maintaining phage resistance (28). Our results demonstrated that despite minor fitness trade-offs, both phage resistance strategies preserved recombinant protein yields comparable to those of the wild-type strain (Fig. 6F). The protein production levels of T7-driven DNA helicases did not significantly differ among the wild-type, spontaneously mutated phage-resistant, and engineered CRISPR/Cas9 recombinant strains. This finding is crucial, as it suggests that introducing phage resistance mechanisms does not compromise bioproduction efficiency. This contrasts with other reports where genetic modifications resulted in reduced metabolic efficiency (29, 30). The stability of protein production in our study may be attributed to the relatively low metabolic burden of CRISPR/Cas9 maintenance and the adaptability of BL21(DE3) as a robust expression host.

This study systematically compared spontaneous mutation and CRISPR/Cas9-mediated immunity as two strategies for engineering phage-resistant *E. coli* strains. Spontaneous mutation provides broad-spectrum resistance but at the cost of fitness and evolutionary stability, whereas CRISPR/Cas9 ensures long-term, programmable immunity with minimal growth defects (Fig. 4B). Importantly, both strategies successfully protected bacterial cultures from phage infection without compromising recombinant protein production, highlighting their potential for industrial applications. Given the trade-offs between stability, specificity, and metabolic burden, future research should focus on integrating multiple defense mechanisms to develop next-generation phage-resistant microbial platforms. This may include combinations of CRISPR/Cas9 with receptor modifications or leveraging anti-CRISPR inhibitors to counteract phage adaptation, ensuring sustainable and efficient fermentation processes.

Biological characterization of the phage TR2 in this study was performed on the basis of empirical comparisons, and the results should be interpreted as relative optimal values under specific experimental conditions rather than absolute universal standards. Moreover, notably, the CRISPR/Cas9 system constructed in this study is based on the commercially available *E. coli* BL21(DE3) strain, which is widely used in synthetic biology and protein expression research because of its robust genetic manipulability and experimental controllability. In this context, the introduction of an exogenous plasmid encoding the CRISPR/Cas9 system does not substantially alter the fundamental biological characteristics of this particular host strain. However, this method—when applied to other bacterial hosts—may result in recombinant strains carrying exogenous functional elements that are classified as genetically modified organisms (GMOs), thereby subject to more stringent regulatory oversight. Therefore, to ensure the broader applicability of this system, future studies should further evaluate its biosafety and consider current bioengineering ethics and regulatory frameworks in accordance with practical application requirements.

## MATERIALS AND METHODS

### Bacterial culture conditions

*E. coli* BL21(DE3) was incubated overnight in LB broth at 37°C with shaking. A total of 27 different bacterial strains were used for the host range assay.

### Phage isolation and purification

We used *E. coli* BL21(DE3) as the host to isolate phages from contaminated fermentation substrate following the method described by Marei (31). In brief, 1 mL of fermentation substrate was centrifuged at 8,000 × *g* for 3 min, and then the supernatant was filtered with a 0.22 µm filter (JINTENG Ltd., Tianjin, China) to remove bacterial debris. A mixture of 100 µL of bacterial culture in the exponential growth phase and 1 mL of fermentation substrate filtrate was incubated in LB broth at 37°C with shaking for 6 h. After incubation, the culture was filtered through a 0.22 µm filter, and the resulting filtrate was diluted with phosphate-buffered saline (PBS). Two-microliter aliquots of the diluted samples were dropped onto the bacterial lawn of double-layer agar plates and incubated at 37°C for 12 h to observe phage plaque formation. Single clear and transparent phage plaques were selected and incubated again. The purification process was repeated three to five times to obtain consistent plaque morphology. Purified plaques were inoculated into 5 mL of bacterial culture in the exponential growth phase and incubated at 37°C for 6 h. Then, the culture was filtered and stored. Phage titers were determined using the double-layer plate method (32).

### Transmission electron microscopy

As described by Feyereisen et al. (33), the morphology of phage TR2 was observed using transmission electron microscopy. Briefly, 10 µL of high-titer phage solution (ca. 10^10^ PFU/mL) was dropped onto a copper grid and allowed to adsorb for 15 min. Excess liquid was blotted dry with filter paper, and the adsorbed samples were subsequently negatively stained with 10 µL of 2% phosphotungstic acid for 2 min. Phage morphology was examined using a JEM-1200EX transmission electron microscope (TEM) (JEOL Ltd., Tokyo, Japan).

### Phage genomic DNA extraction, sequencing, and bioinformatics analysis

Phage DNA was extracted using the λ Phage Genome Extraction Kit (Leagene Biotechnology Ltd., Beijing, China). The DNA library was prepared using the NEBNext Ultr II DNA Library Prep Kit for Illumina (New England Biolabs Ltd., Beijing, China) and sequenced using the NovaSeq 6000 platform (Illumina, San Diego, United States) with the NovaSeq 6000 S4 Reagent Kit v1.5 (Illumina, San Diego, United States) using a PE150 sequencing strategy. Low-quality reads were filtered using Trimmomatic (V0.32) (34), and the complete genome of phage TR2 was assembled with SPAdes v3.13.0 (35). Finally, the genome was visualized using Bandage software (36). A Bacterial Genomic DNA Extraction Kit (Solarbio, Beijing, China) was used to extract the bacterial genome following the same sequencing strategy used for the phage.

Open reading frames (ORFs) within the phage genome were predicted using the online database RAST (https://rast.nmpdr.org/) (37), and all annotations were verified by NCBI BLASTp software (https://blast.ncbi.nlm.nih.gov/Blast.cgi). tRNAs were predicted using the tRNAscan-SE Search Server (http://lowelab.ucsc.edu/tRNAscan-SE/) (38). A circular genome map was constructed using the CGView Server (http://www.bioinformatics.org/cgview/gallery.html) (39). Virulence factor genes and antibiotic resistance genes were predicted using the online tool Center for Genomic Epidemiology (http://www.genomicepidemiology.org/services/) and the Virulence Factor Database (VFDB) (http://www.mgc.ac.cn/VFs/) (40). The analysis of VFDB can be performed using the online platform or locally using the diamond software to align the downloaded protein sequences with the VFDB database, thereby obtaining the relevant annotation results. Phylogenetic tree construction and sequence similarity analysis were performed using VICTOR (https://ggdc.dsmz.de/victor.php) (41) and VIRIDIC (http://rhea.icbm.uni-oldenburg.de/VIRIDIC/) (42). Comparative genome analysis was conducted using the EasyFig 2.2.3 program (43), and the PhageTerm tool was used to predict the phage genome termini type and packaging mechanism (44).

### Characterization of phage TR2

#### Host range

The EOP of phage TR2 against 27 different bacterial strains was determined as described by Dong et al. (45). Initially, 2 µL of a serial tenfold dilution of the phage mixture, ranging from 10^1^ to 10^8^ PFU/mL, was dropped onto the bacterial lawn of double-layer plates. Phage plaque formation was observed after incubation at 37°C overnight to assess the sensitivity of different strains to phage TR2.

#### Optimal MOI value

To determine the optimal MOI for phage TR2, *E. coli* BL21(DE3) cultures at their peak growth phase were mixed with phage TR2 in LB broth at MOIs of 0.01, 0.1, 1, and 10. The mixture was incubated at 37°C with shaking for 6 h. Subsequently, the cultures were sampled, and the phage titer was quantified using the double-layer agar method. The optimal MOI was determined on the basis of the highest phage yield (46).

#### One-step growth curve

A one-step growth curve was generated to determine the latency period and burst size of phage TR2. Exponentially growing *E. coli* BL21(DE3) cells were infected with phage TR2 at the optimal MOI. After phage adsorption for 10 min, the mixture was centrifuged at 8,000 × *g* for 1 min, and the supernatant was discarded. The precipitate was then washed with PBS to remove unadsorbed phages. The resulting suspension was incubated at 37°C for 180 min, with samples taken every 10 min for phage titer determination using the double-layer agar method.

#### Adsorption curve

The adsorption kinetics of phage TR2 were assessed by mixing 1 mL of *E. coli* BL21(DE3) in the exponential phase with 1 mL of phage TR2 (ca. 1 × 10^6^ PFU/mL). Samples were collected every 2.5 min and immediately filtered to remove and quantify unabsorbed phages using the double-layer agar method. The percentage of adsorbed phages was calculated, and the adsorption over time was plotted (47).

#### Thermal stability

To evaluate the thermal stability of phage TR2, 1 mL of phage TR2 (ca. 3 × 10^8^ PFU/mL) was placed at various temperatures: 4°C, 20°C, 37°C, 50°C, 65°C, and 80°C. Phage viability was measured after incubation for 0.5 h, 1 h, and 2 h.

#### pH stability

For the pH stability assessment, 50 µL of phage TR2 (ca. 3 × 10^8^ PFU/mL) was mixed with 950 µL of PBS at various pH values ranging from 2 to 13. The surviving phages were quantified using the double-layer agar method after incubation at 37°C for 1 h and 2 h.

### Phage-resistant strain preparation and mutation analysis

The phage-resistant *E. coli* strain was prepared by incubating 100 µL of BL21(DE3) in the exponential growth phase with 1 mL of high-titer phage TR2 (approximately 10^8^ pfu/mL) in 5 mL of LB broth at 37°C for 24 h. The culture was streaked onto LB agar inoculated with the phage to obtain spontaneously mutated phage-resistant strains (Fig. 4A). Mutations in spontaneously mutated phage-resistant strains were analyzed using the breseq v0.37.1 tool (48) with *E. coli* BL21(DE3) as the reference strain and identified by comparison with the wild-type strain (49).

### Construction of recombinant BL21(DE3)

Two CRISPR/Cas9-engineered recombinant strains were constructed by introducing plasmids that express the CRISPR/Cas9 system, which consists of the Cas9 nuclease and a chimeric single-guide RNA (sgRNA), into *E. coli* BL21(DE3) via electroporation. The strain carrying a spacer targeting ORF68 of phage TR2, which encodes the major capsid protein (5′-TGGTCTGGCTCTGCACCTCA-3′), was designated BL21(DE3)-C. The strain targeting ORF72, which encodes the large terminase subunit (5′-GAGGTTTGAATCGATATCTA-3′), was designated BL21(DE3)-T. The spacer sequences were designed using the specificity score calculation tool (http://crispr.dfci.harvard.edu/SSC/).

### Antiphage activities of phage-resistant strains

The antiphage activities of the spontaneously mutated phage-resistant strains and engineered CRISPR/Cas9 recombinant strains prepared in our previous work (45) were evaluated using the EOP method. All phage-resistant strains were tested against 10 *E. coli* phages obtained from Tonglab Phage Library. Double-layer plates containing bacteria in the exponential growth phase were prepared, with gentamicin (20 µg/mL) and arabinose (0.2%) added to LB soft agar for the CRISPR/Cas9 recombinant strains. A serial tenfold dilution of phage solution (initial titer of 1 × 10^8^ PFU/mL) was then inoculated onto the plates. After overnight incubation at 37°C, phage plaque formation was recorded. Fold protection was evaluated by comparing the EOP of phages on the BL21(DE3) strain with the EOP on spontaneously mutated phage-resistant strains and engineered CRISPR/Cas9 recombinant strains.

### Bacterial growth curves

Bacterial growth curves were generated as described by Zou et al. (28). One hundred microliters of a 1 × 10^8^ CFU/mL bacterial culture and 100 µL of phage solution were inoculated into 5 mL of LB broth at various MOIs. Gentamicin and arabinose were added to LB broth for incubation of the recombinant CRISPR/Cas9 strains. The OD_600_ of the culture was measured every hour using a microplate reader (Thermo Fisher Scientific, Beijing, China) to monitor bacterial growth.

### Protein expression

The plasmid pET28a containing the DNA helicase gene was introduced into BL21(DE3) competent cells via electroporation and incubated in 800 mL of LB broth supplemented with 50 µg/mL kanamycin (final concentration). Gentamicin and arabinose were added to LB broth for incubation of the recombinant CRISPR/Cas9 strains. When the culture reached an OD_600_ of 0.6, IPTG was added at a final concentration of 0.5 mM to induce expression, and the culture was grown at 16°C for 20 h. Then, the culture was centrifuged and harvested. The protein concentration of each sample was determined by measuring the OD_280_ using a NanoDrop One microvolume spectrophotometer (Thermo Scientific) to standardize the loading quantity for SDS‒PAGE analysis. DNA helicase expression was assessed using Coomassie Brilliant Blue staining and western blotting. A gel imager (Tanon, Shanghai, China) was used to visualize the results. Quantitative analysis of the western blot results was performed using ImageJ software. The relative molecular weight of the DNA helicase protein was predicted by ExPASy (http://www.expasy.org).

### Stability of phage resistance

The experimental design for evaluating the stability of phage resistance is depicted in Fig. 5A. Initially, 1 mL of wild-type phage (1 × 10^9^ PFU/mL) was cocultured with 1 mL of either spontaneously mutated phage-resistant strains or engineered CRISPR/Cas9 recombinant strains (1 × 10^8^ PFU/mL) in 100 mL of LB broth at 37°C for five days. After this period, 1 mL of the coculture was filtered to collect the phage, and this phage suspension was labeled the first-round culture. Subsequently, 1 mL of the first-round culture was combined with the bacterial suspension under identical conditions to commence a new round of stability evaluation. The experiment was conducted over three cycles. For BL21(DE3)-N-1, since the wild-type phage TR2 could not form plaques on the plates inoculated with BL21(DE3)-N-1, the formation of phage plaques served as an indicator of phage escape from bacterial defense. For the other three strains, phage titers in the culture medium were initially determined, followed by coinoculation with wild-type phage of the same titer on plates of the corresponding strain. Phage escape from bacterial defenses was assessed on the basis of changes in the efficiency of plaque formation.

### Statistical analysis

All the data are presented as the means ± standard deviations (SDs). Statistical analyses were performed using one-way analysis of variance (ANOVA) followed by Tukey’s multiple comparison test in GraphPad Prism version 10.0.2. A *P* value of less than 0.05 was considered indicative of statistical significance. Statistical significance is indicated as follows: *P < 0.05, **P < 0.01, ***P < 0.001, and ****P < 0.0001.

The data in Fig. 2 and Fig. S1 were visualized using the online platform Bioinformatics (https://www.bioinformatics.com.cn/), while all other figures were plotted with Origin 2021.

## Data Availability

The complete genome sequence of phage TR2 has been deposited in GenBank under the accession number OP251154.1.

## SUPPLEMENTAL MATERIAL

Fig. S1 aem.01596-25-s0001.tif Biological characteristics of phage TR2.

Fig. S2 aem.01596-25-s0002.tif Kinetic curve of phage adsorption.

Fig. S3 aem.01596-25-s0003.tif Analysis of recombinant protein expression and detection.

Supplemental tables aem.01596-25-s0004.docx Legends for Fig. S1 to S3; Tables S1 and S2.

ASM does not own the copyrights to Supplemental Material that may be linked to, or accessed through, an article. The authors have granted ASM a non-exclusive, world-wide license to publish the Supplemental Material files. Please contact the corresponding author directly for reuse.
