# Supplementary figures and images for: Engineering broad-spectrum phage-resistant Escherichia coli via adaptive and programmable defense strategies
Source: Appl Environ Microbiol. 2025 Oct 22;91(11):e01596-25. doi: 10.1128/aem.01596-25 (PMC12628846; doi:10.1128/aem.01596-25)

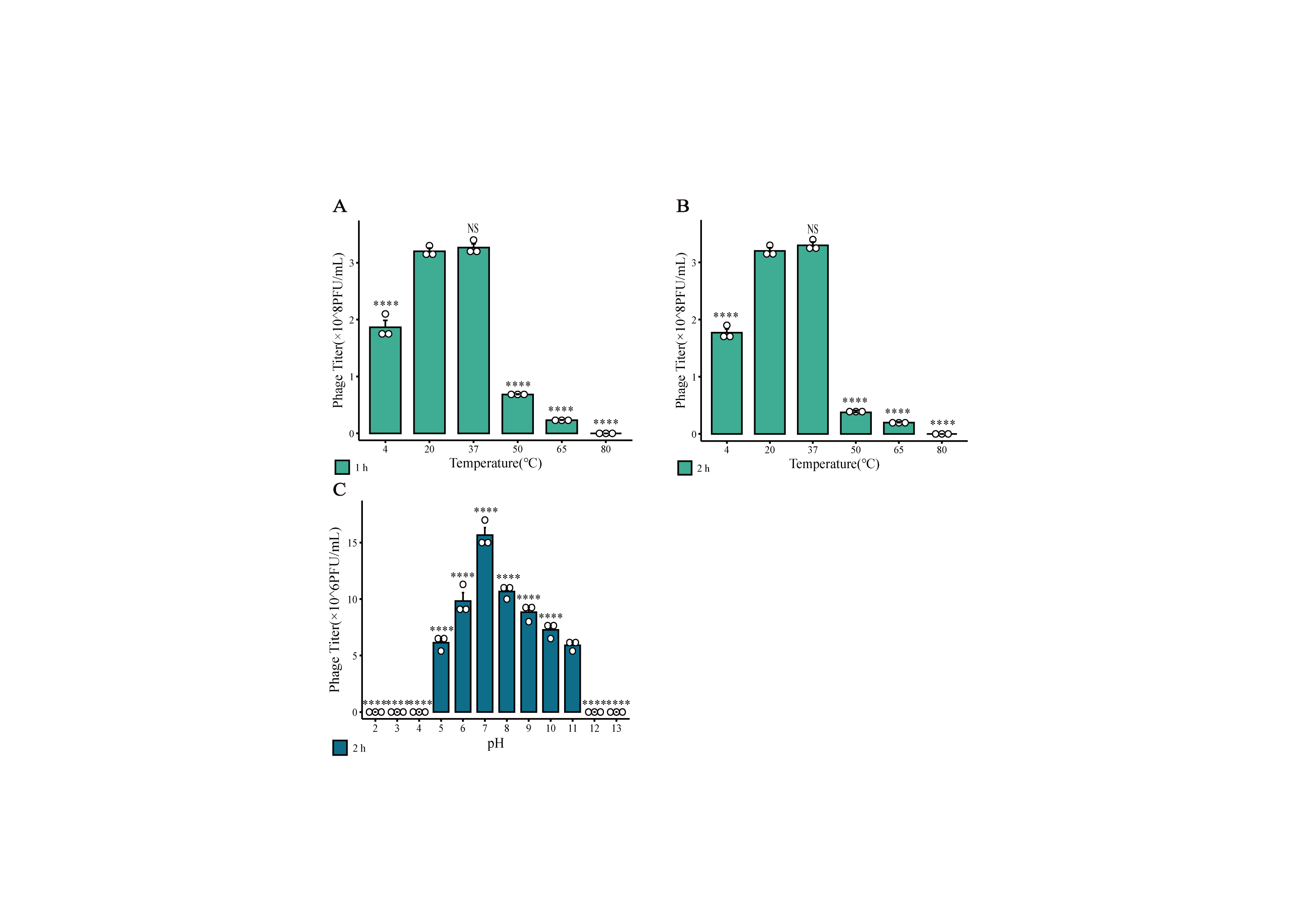

Supplement: Fig. S1 — Biological characteristics of phage TR2. [file aem.01596-25-s0001.tif]

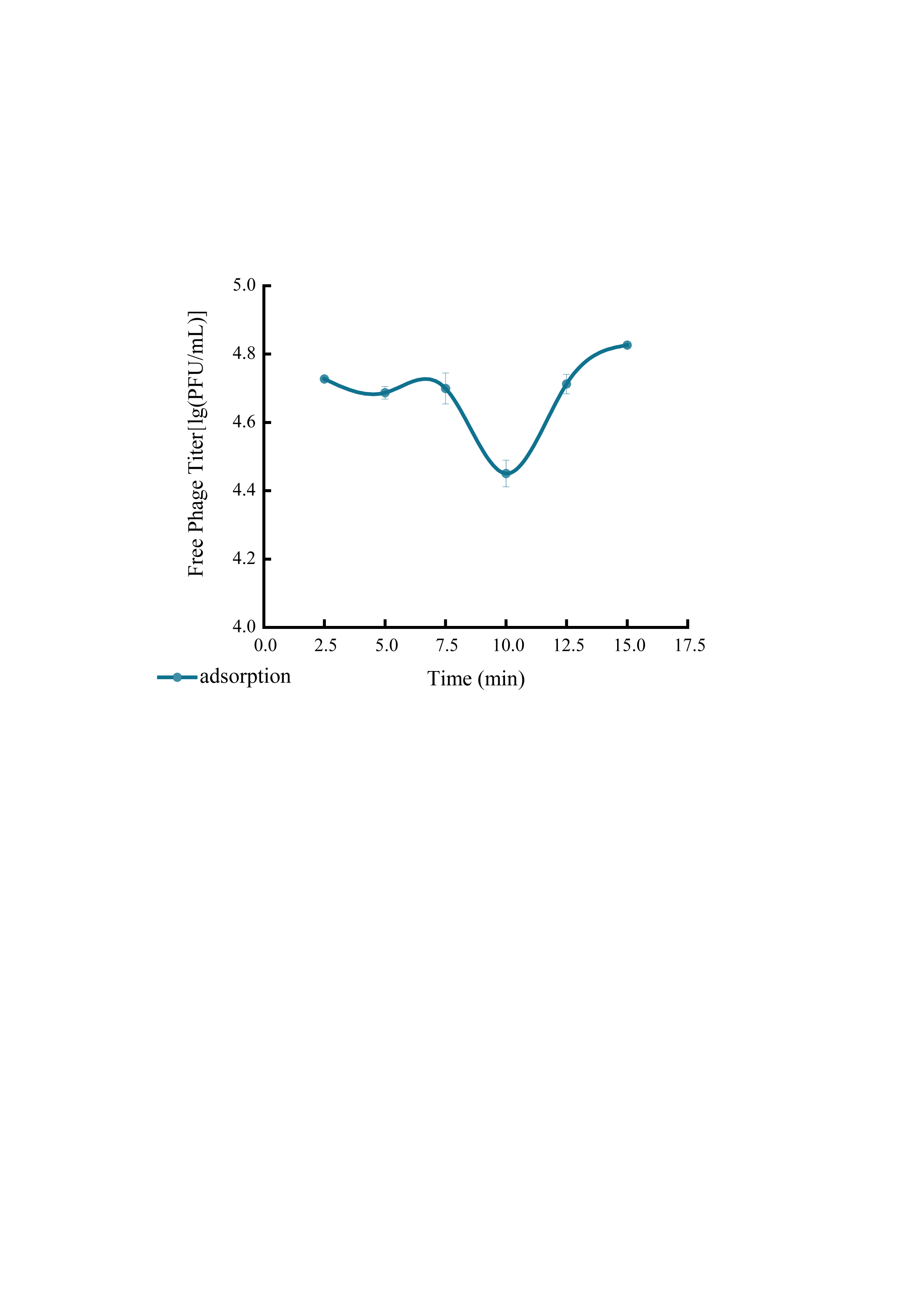

Supplement: Fig. S2 — Kinetic curve of phage adsorption. [file aem.01596-25-s0002.tif]

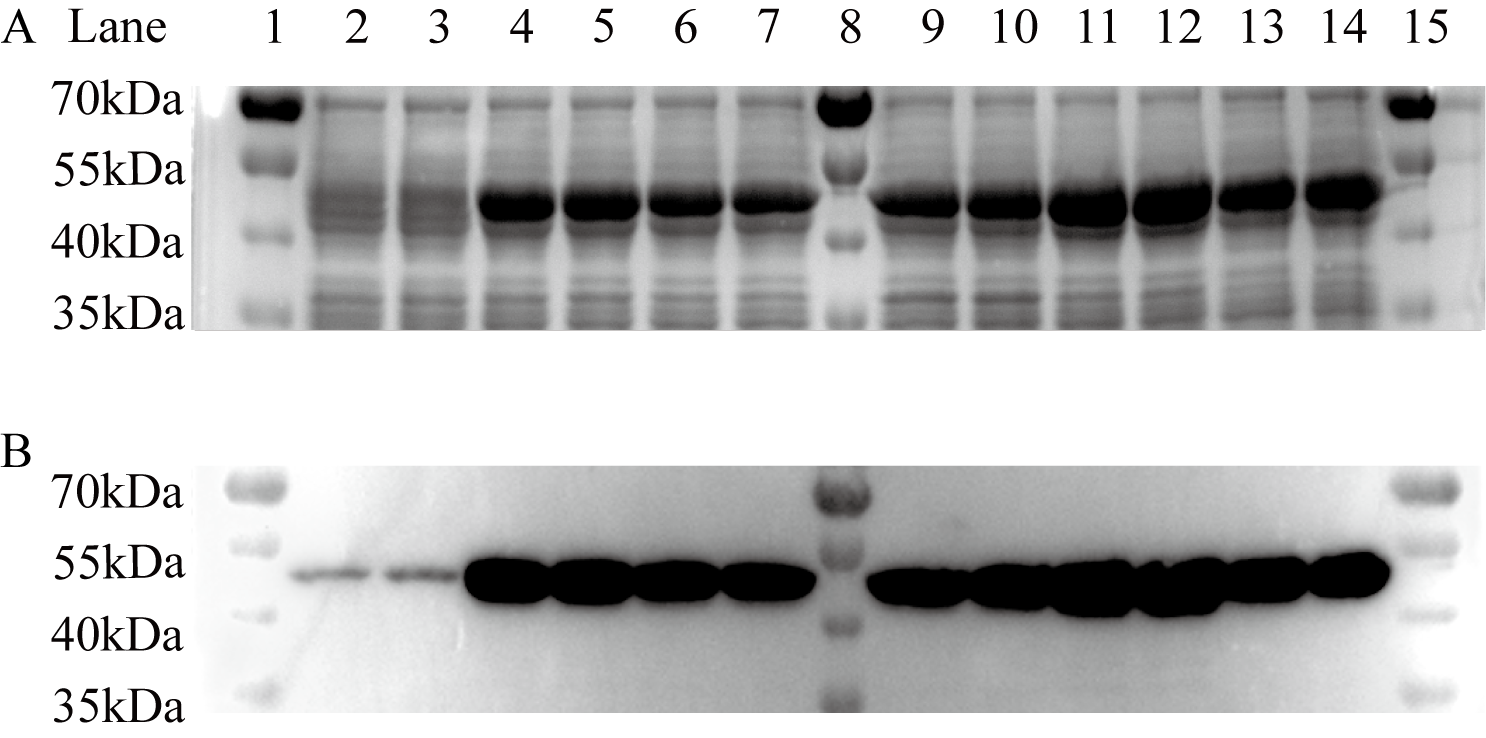

Supplement: Fig. S3 — Analysis of recombinant protein expression and detection. [file aem.01596-25-s0003.tif]
