# Supplementary material for: Engineering broad-spectrum phage-resistant Escherichia coli via adaptive and programmable defense strategies
Source: Appl Environ Microbiol. 2025 Oct 22;91(11):e01596-25. doi: 10.1128/aem.01596-25 (PMC12628846; doi:10.1128/aem.01596-25)
Supplement: Supplemental tables — Legends for Fig. S1 to S3; Tables S1 and S2. [file aem.01596-25-s0004.docx]

**Fig S1** Biological characteristics of phage TR2. (A): The thermal stability of phage TR2 has been assessed over a duration of 1 hour. (B): The thermal stability of phage TR2 has been assessed over a duration of 2 hours. (C): The pH Stability of phage TR2 has been assessed over a duration of 2 hours.

**Fig S2** Kinetic curve of phage adsorption.

**Fig S3** Analysis of recombinant protein expression and detection. (A) Coomassie Brilliant Blue staining of total cellular proteins. Lane 1,8,15: Protein molecular weight marker; Lane 2,3: Uninduced control; Lane 4,5: Induced BL21(DE3); Lane 6,7: Induced BL21(DE3)-N-1. Lane 9,10: Induced BL21(DE3)-N-2. Lane 11,12: Induced BL21(DE3)-C. Lane 13,14: Induced BL21(DE3)-T. The anticipated molecular weight of the target protein is 49.2 kDa. (B) Western blot analysis of recombinant protein expression. Proteins were probed with anti-His tag antibody (1:1000 dilution). Lane assignments are identical to those in (A).

Table S1: The host range of phage TR2

| Strain | Species | Infectivity | Description |
| --- | --- | --- | --- |
| BL21(DE3） | *Escherichia coli* | + | Beijing University of Chemical Technology - Phage Research Center |
| DH5α | *Escherichia coli* | + |  |
| 3101 | *Staphylococcus aureus* | - | General Hospital of the Chinese People's Liberation Army |
| 3107 | *Staphylococcus aureus* | - |  |
| 3111 | *Staphylococcus aureus* | - |  |
| 3955 | *Escherichia coli* | - | Qingyang People's Hospital |
| 3964 | *Escherichia coli* | - |  |
| 3965 | *Enterobacter cloacae* | - |  |
| 3966 | *Escherichia coli* | - |  |
| 3980 | *Escherichia coli* | - |  |
| 3982 | *Enterobacter cloacae* | - |  |
| 3997 | *Escherichia coli* | - |  |
| 3998 | *Escherichia coli* | - |  |
| 4012 | *Escherichia coli* | - |  |
| 4013 | *Escherichia coli* | - |  |
| 4014 | *Escherichia coli* | - |  |
| 4015 | *Escherichia coli* | - |  |
| 4072 | ***Proteus mirabilis*** | - |  |
| 4099 | ***Proteus mirabilis*** | - |  |
| 4259 | ***Klebsiella pneumoniae*** | - | Seventh Medical Centre |
| 4263 | ***Klebsiella pneumoniae*** | - |  |
| 4270 | ***Klebsiella pneumoniae*** | - |  |
| 4281 | *Pseudomonas aeruginosa* | - |  |
| 4291 | *Pseudomonas aeruginosa* | - |  |
| 4298 | *Pseudomonas aeruginosa* | - |  |
| 4300 | *Acinetobacter baumannii* | - |  |
| 4303 | *Acinetobacter baumannii* | - |  |

Note: + lysis, - without lysis.

Table S2: Phage TR2 open reading frame information

| NO. | Start | Stop | Predicted Function | Best match | Accession number | E value | Query Cover | Per.Identity |
| --- | --- | --- | --- | --- | --- | --- | --- | --- |
| 1 | 305 | 123 | DUF3667 domain-containing protein | *Escherichia* phage IMM-001 | ATI17072.1 | 5.00E-36 | 100% | 96.67% |
| 2 | 397 | 325 | tRNA-Arg |  |  |  |  |  |
| 3 | 833 | 597 | Transcriptional repressor | *Escherichia* phage IMM-001 | ATI17068.1 | 1.00E-44 | 97% | 90.79% |
| 4 | 1062 | 826 | Hypothetical protein | *Escherichia* phage vB_EcoS_IME542 | YP_009824909.1 | 5.00E-35 | 100% | 96.15% |
| 5 | 1277 | 1119 | Hypothetical protein | *Escherichia* phage vB_EcoD_SU57 | QLF85036.1 | 1.00E-15 | 84% | 70.45% |
| 6 | 1428 | 1270 | Hypothetical protein | *Escherichia* phage IMM-001 | ATI17064.1 | 6.00E-16 | 78% | 78.05% |
| 7 | 1581 | 1447 | Hypothetical protein | *Escherichia* phage vB_EcoS_MM01 | QBQ80840.1 | 1.00E-11 | 97% | 58.14% |
| 8 | 1771 | 1592 | Hypothetical protein | *Escherichia* phage ZL19 | UMO77922.1 | 2.00E-34 | 100% | 94.92% |
| 9 | 1977 | 1768 | Methyltransferase | *Escherichia* phage IMM-001 | ATI17060.1 | 7.00E-31 | 100% | 76.81% |
| 10 | 2531 | 2049 | Hypothetical protein | *Escherichia* phage vB_EcoD_SU57 | QLF85048.1 | 2.00E-74 | 100% | 71.88% |
| 11 | 3052 | 2573 | Hypothetical protein | *Escherichia* phage ZL19 | UMO77910.1 | 2.00E-80 | 100% | 83.02% |
| 12 | 3521 | 3393 | Hypothetical protein | *Escherichia* phage IMM-001 | ATI17045.1 | 3.20E-02 | 59% | 68.00% |
| 13 | 3727 | 3924 | Hypothetical protein | Caudovirales sp. | DAH81959.1 | 2.00E-17 | 72% | 74.47% |
| 14 | 3921 | 4115 | Hypothetical protein | *Escherichia* phage vB_EcoD_SU57 | QLF85004.1 | 2.00E-13 | 96% | 50.79% |
| 15 | 4117 | 4320 | Hypothetical protein | *Escherichia* phage UPEC06 | QUL77167.1 | 4.00E-11 | 98% | 37.88% |
| 16 | 4320 | 4481 | Hypothetical protein | *Escherichia* phage vB_EcoD_SU57 | QLF85005.1 | 1.00E-30 | 100% | 96.23% |
| 17 | 4541 | 4705 | Hypothetical protein | *Escherichia* phage vB_EcoS_MM01 | QBQ80900.1 | 1.00E-16 | 98% | 92.45% |
| 18 | 4705 | 5058 | Hypothetical protein | *Escherichia* phage vB_EcoD_SU57 | QLF85007.1 | 2.00E-68 | 100% | 85.47% |
| 19 | 5132 | 6715 | DUF3987 domain-containing protein | *Escherichia* phage vB_EcoS_IME542 | YP_009824889.1 | 0.00E+00 | 100% | 96.96% |
| 20 | 6719 | 7054 | Hypothetical protein | *Escherichia* phage vB_EcoS_IME542 | YP_009824888.1 | 4.00E-71 | 100% | 91.89% |
| 21 | 7452 | 7066 | U-spanin | *Escherichia* phage JeanPiccard | QXV80817.1 | 1.00E-48 | 93% | 71.54% |
| 22 | 7934 | 7449 | Lysozyme | *Escherichia* phage vB_EcoS_ACG-M12 | YP_006987885.1 | 4.00E-87 | 98% | 85.53% |
| 23 | 8149 | 7934 | Holin | *Escherichia* phage vB_EcoS_IME542 | YP_009824885.1 | 2.00E-22 | 100% | 85.92% |
| 24 | 8451 | 8278 | Hypothetical protein | *Escherichia* phage BEK6 | QGH76894.1 | 1.00E-27 | 100% | 80.70% |
| 25 | 9064 | 8510 | ATPase | *Escherichia* phage vB_EcoD_SU57 | QLF84977.1 | 5.00E-101 | 99% | 75.96% |
| 26 | 9662 | 9114 | Polynucleotide kinase | *Escherichia* phage P818 | UOX38508.1 | 8.00E-92 | 98% | 72.63% |
| 27 | 10873 | 9740 | DNA binding protein | Caudovirales sp. | DAF69443.1 | 0.00E+00 | 100% | 86.21% |
| 28 | 11206 | 10955 | Hypothetical protein | *Escherichia* phage vB_EcoD_SU57 | QLF85014.1 | 3.00E-51 | 98% | 92.68% |
| 29 | 11451 | 11209 | Hypothetical protein | *Escherichia* phage vB_EcoS_ACG-M12 | YP_006987877.1 | 4.00E-45 | 98% | 86.08% |
| 30 | 11573 | 11448 | Hypothetical protein | *Escherichia* phage vB_EcoD_SU57 | QLF85015.1 | 8.00E-18 | 100% | 85.37% |
| 31 | 11794 | 11573 | Hypothetical protein | *Enterobacteria* phage vB_EcoS_Rogue1 | YP_007112254.1 | 4.00E-38 | 94% | 88.41% |
| 32 | 11994 | 11794 | Hypothetical protein | *Escherichia* phage vB_EcoS_FP | QLF80597.1 | 5.00E-34 | 100% | 87.88% |
| 33 | 12492 | 12073 | Nuclease | Caudovirales sp. | DAH81930.1 | 3.00E-93 | 100% | 92.81% |
| 34 | 14483 | 12489 | DNA helicase | *Escherichia* phage AugustePiccard | QXV76173.1 | 0.00E+00 | 100% | 92.32% |
| 35 | 14580 | 15062 | Transcription regulator | *Escherichia* phage vB_EcoS_ESCO41 | YP_009789963.1 | 3.00E-89 | 88% | 88.73% |
| 36 | 15129 | 15653 | HNH endonuclease | *Pectobacterium* phage vB_PatM_CB7 | ARB11665.1 | 5.00E-32 | 96% | 41.42% |
| 37 | 15650 | 16573 | DNA primase | *Escherichia* phage vB_EcoD_SU57 | QLF84982.1 | 0.00E+00 | 99% | 82.30% |
| 38 | 16659 | 18155 | Lateral tail protein | *Escherichia* phage AugustePiccard | QXV76169.1 | 1.00E-69 | 29% | 85.81% |
| 39 | 18604 | 18185 | Single-stranded DNA binding protein | *Escherichia* phage JeanPiccard | QXV80799.1 | 1.00E-65 | 77% | 88.89% |
| 40 | 19295 | 18645 | Recombinase | *Escherichia* phage IMM-001 | ATI17130.1 | 6.00E-144 | 100% | 89.81% |
| 41 | 19844 | 19365 | HNH endonuclease | *Escherichia* phage vB_EcoS_IME542 | YP_009824939.1 | 3.00E-54 | 99% | 55.70% |
| 42 | 20890 | 19919 | Exonuclease | *Escherichia* phage IMM-001 | ATI17126.1 | 0.00E+00 | 100% | 85.45% |
| 43 | 21426 | 20932 | Regulatory protein | Caudovirales sp. | DAH81905.1 | 2.00E-62 | 100% | 58.54% |
| 44 | 21625 | 21428 | Hypothetical protein | *Escherichia* phage IMM-001 | ATI17124.1 | 2.00E-33 | 96% | 87.30% |
| 45 | 22037 | 21630 | Hypothetical protein | *Escherichia* phage JeanPiccard | QXV80793.1 | 4.00E-29 | 81% | 48.65% |
| 46 | 22452 | 22589 | KilA protein | *Escherichia* phage vB_EcoS-IME253 | YP_009789229.1 | 7.00E-22 | 100% | 93.33% |
| 47 | 22692 | 23129 | DNA-binding protein | *Escherichia* phage vB_EcoS_CEB_EC3a | YP_009789335.1 | 4.00E-69 | 88% | 80.62% |
| 48 | 23186 | 23446 | Hypothetical protein | *Escherichia* virus ECH1 | QFP93005.1 | 5.00E-51 | 98% | 91.76% |
| 49 | 23515 | 23757 | Lipoprotein | *Escherichia* phage vB_EcoS_CEB_EC3a | YP_009789334.1 | 2.00E-40 | 100% | 88.75% |
| 50 | 23757 | 24713 | Tail tip protein | *Escherichia* phage vB_EcoS_IME542 | YP_009824935.1 | 3.00E-147 | 100% | 68.75% |
| 51 | 28128 | 24742 | Tail fiber protein | *Escherichia* phage IMM-001 | ATI17114.1 | 0.00E+00 | 100% | 94.95% |
| 52 | 28783 | 28208 | Tail assembly protein | *Escherichia* phage IMM-001 | ATI17112.1 | 9.00E-108 | 100% | 84.77% |
| 53 | 29423 | 28767 | Minor tail protein | *Escherichia* phage IMM-001 | ATI17111.1 | 3.00E-155 | 100% | 94.04% |
| 54 | 29787 | 29578 | Hypothetical protein | *Escherichia* phage SZH-1 | URG18158.1 | 1.00E-05 | 88% | 42.62% |
| 55 | 30615 | 29860 | Minor tail protein | *Escherichia* phage BEK6 | QGH76866.1 | 2.00E-160 | 100% | 83.27% |
| 56 | 31004 | 30654 | Minor tail protein | *Escherichia* phage IMM-001 | ATI17108.1 | 1.00E-79 | 100% | 96.55% |
| 57 | 34008 | 31036 | Tail type measure protein | *Escherichia* phage vB_EcoS_CEB_EC3a | YP_009789327.1 | 0.00E+00 | 100% | 69.62% |
| 58 | 34297 | 34046 | TfmS | *Escherichia* phage IMM-001 | ATI17106.1 | 2.00E-54 | 100% | 97.59% |
| 59 | 34674 | 34360 | Tail assembly chaperone | *Escherichia* phage IMM-001 | ATI17105.1 | 8.00E-70 | 100% | 100.00% |
| 60 | 34958 | 34710 | Hypothetical protein | *Escherichia* phage IMM-001 | ATI17102.1 | 2.00E-42 | 100% | 84.34% |
| 61 | 35890 | 35231 | Major capsid protein | *Escherichia* phage vB_EcoD_SU57 | QLF84993.1 | 1.00E-133 | 99% | 88.53% |
| 62 | 36300 | 35902 | Minor tail protein | *Escherichia* phage IMM-001 | ATI17099.1 | 2.00E-92 | 100% | 98.48% |
| 63 | 36737 | 36300 | Hypothetical protein | *Escherichia* phage IMM-001 | ATI17098.1 | 2.00E-91 | 100% | 91.03% |
| 64 | 37101 | 36730 | Hypothetical protein | *Escherichia* phage IMM-001 | ATI17096.1 | 6.00E-80 | 100% | 97.56% |
| 65 | 37493 | 37098 | Hypothetical protein | *Escherichia* phage IMM-001 | ATI17094.1 | 4.00E-71 | 100% | 87.02% |
| 66 | 37780 | 37535 | Hypothetical protein | *Escherichia* phage vB_EcoS_IME542 | YP_009824919.1 | 1.00E-46 | 100% | 91.36% |
| 67 | 38407 | 37811 | HNH endonuclease | *Shigella* phage Sd1 | YP_009791941.1 | 4.00E-28 | 92% | 37.50% |
| 68 | 39423 | 38479 | Major capsid protein | *Escherichia* phage AugustePiccard | QXV76142.1 | 0.00E+00 | 100% | 92.04% |
| 69 | 40060 | 39565 | Hypothetical protein | *Escherichia* phage vB_EcoD_SU57 | QLF85029.1 | 5.00E-86 | 100% | 78.16% |
| 70 | 41184 | 40072 | Prohead protease | *Escherichia* phage 2725-N35 | QHJ72673.1 | 0.00E+00 | 99% | 82.83% |
| 71 | 42448 | 41174 | Portal protein | *Escherichia* phage vB_EcoS_FP | QLF80552.1 | 0.00E+00 | 99% | 76.30% |
| 72 | 43940 | 42498 | Terminase large subunit | *Escherichia* phage vB_EcoS_FP | QLF80551.1 | 0.00E+00 | 100% | 94.17% |
| 73 | 44578 | 44072 | HNH endonuclease | *Salmonella* phage Segz_1 | YP_010053381.1 | 2.00E-37 | 94% | 46.84% |
| 74 | 45171 | 44647 | Terminase small subunit | *Escherichia* phage IMM-001 | ATI17076.1 | 7.00E-118 | 100% | 92.53% |

Note: +, - are used to indicate the direction of transcription.
